# Physiological Gingival Melanin Hyperpigmentation Treatment with Injectable Vitamin C and Scalpel Technique: A Randomised Controlled Clinical Trial

**DOI:** 10.1155/2023/4586923

**Published:** 2023-01-18

**Authors:** Dhanashree S. Chaudhary, Simran R. Parwani, Shital Barkhade, Minal Gajbhiye, Rajkumar Parwani, Geetanjali Sikka, Kshipra Kawadkar, Nishita Jaju Soni, Niccolò Giuseppe Armogida, Himanshu Dadlani, Gianrico Spagnuolo

**Affiliations:** ^1^Department of Periodontology, V.Y.W.S. Dental College and Hospital, Amravati, Maharashtra 444602, India; ^2^Department of Oral Pathology, V.Y.W.S. Dental College and Hospital, Amravati, Maharashtra 444602, India; ^3^Department of Periodontology, Eklavya Dental College & Hospital Kotpulti, Rajasthan 303108, India; ^4^Department of Neurosciences, Reproductive and Odontostomatological Sciences, University of Naples Federico II, Naples 80131, Italy; ^5^Department of Periodontology, Kalka Dental College, Meerut 250002, India; ^6^Kalka Dental College, Meerut 250016, India; ^7^Therapeutic Dentistry Department, Institute for Dentistry, Sechenov University, Moscow 119991, Russia

## Abstract

Harmony between facial complexion and gingival health goes hand in hand. Gingival depigmentation is an aesthetic correction of hyperactive melanocytes in gingival tissues that lead to hyperpigmentation. Current study compares depigmentation, pain scores, and itching with scalpel technique and nonsurgical intramucosal Vitamin C injection. 30 individuals in the age range of 18–40 years conscious of dark gums were randomly allocated to test and control group by lottery method. Thorough Phase I therapy was performed one week before the procedure. Area and intensity of depigmentation were evaluated preoperatively and postoperatively; pain score, itching, and repigmentation percentage were the postoperative parameters. After 24 hrs, test group showed significantly lesser VAS score for pain as compared to control group. There was no statistically significant difference in preoperative area of pigmentation between the test and control group (*p*=0.936). Postoperatively also, there was no statistically significant difference in area of pigmentation between the test and control group (*p*=0.932). For comparing area of pigmentation, an independent *t*-test was applied and Mann–Whitney test was used for differentiating the intensity of pigmentation, repigmentation, and VAS score between the groups. The study concluded that Vitamin C mesotherapy and scalpel technique showed comparable results in reduction of areas and intensity of gingival hyperpigmentation.

## 1. Introduction

Over the last decade, an exponential request of aesthetic treatments has been increasingly observed, especially in the dentistry field. In fact, having an aesthetically satisfying smile plays an important role in the psychological wellbeing and self-confidence. There are various factors that determine the overall aesthetic of smile design such as lip competency, lip line, gums, and teeth morphology [1]. The aesthetic of the gingival tissues, also known as the pink aesthetic, can greatly influence the overall clinical appearance of the smile. Regardless of the color of the gingival tissues which may physiologically vary from pale pink to bluish purple [2], gingival pigmentations may alter and impact the pink aesthetic. Gingival hyperpigmentation can be defined as a darker gingival color beyond what is normally expected and can be attributed to an increased production and release of melanin by the melanocytes in the basal and the suprabasal cells of the epithelium [3]. Melanin is nonhemoglobin-derived brown pigment; its granules set forth normal pigmentation in the skin and oral tissues. Whenever the gingiva is hyperkeratinised there is hyperactivity of melanocytes in the basal layer. That means the activity rather than the number of melanocytes is increased in the basal layers [4, 5].

There are different techniques available for gingival depigmentation, both surgical and nonsurgical. The surgical techniques include split thickness epithelial excision/scalpel technique, electrosurgery, lasers, gingival abrasion/surgical stripping, cryosurgery, or masking by free gingival autografts and acellular dermal matrix allografts. The nonsurgical methods, also called as the gingival peeling techniques, include the use of ascorbic acid (Vitamin C), phenols, salicylic acid, glycolic acid, trichloroacetic acid, and alcohol.

Amidst these, ascorbic acid is the only one that can be handled by controlling the depth of penetration, and it inhibits melanin formation by suppressing the tyrosine activity, which is essential for melanin biosynthesis. Furthermore, ascorbic acid directly downregulates dopaquinone formation, a precursor in melanin synthesis, thus inhibiting melanin formation.

With regards to repigmentation, melanocytes have been successful in repopulating the areas of depigmentation by migration from one site to another or by imparting their products to the surrounding cells by varied communication channels [6]. The length of the dendrites of melanocytes is determined by the number of surrounding keratinocytes. Thicker periodontal phenotypes exhibit greater length and number of melanocyte dendrites for stimulating faster rates of repigmentation [7, 8].

At the present, studies comparing the effectiveness of the surgical and nonsurgical techniques are lacking, and no specific guidelines are available. With the above considerations, the authors carried out a randomised controlled clinical trial with the aim of comparing clinical outcomes of gingival depigmentation following scalpel and injectable Vitamin C techniques. The pain score, itching, and repigmentation were also assessed postoperatively.

## 2. Materials and Methods

### 2.1. Eligibility Criteria for Study Sample

This trial was performed at Periodontology department of VYWS Dental College and Hospital, Amravati, with Institutional Ethical Committee number: DCA/IEC/92/022.

Thirty patients, 18 females (60%) and 12 males (40%) in the age range of 18–40 years who had a chief complaint of pigmented or dark gums were selected. The patients were informed about the study procedure, and an informed consent form was taken. The selection criteria were done according to the pigmentation index by Dummett and Bolden [9].

Gingival pigmentation index is as follows (Dummett and Bolden) [9]:

- Score 0: absence of pigmentation
- Score 1: spots of brown to black color or pigments
- Score 2: brown to black patches but not diffuse pigmentation
- Score 3: diffuse brown to black pigmentation, marginal, and attached

Inclusion criteria included systemically healthy subjects with Dummett and Bolden index score 1, 2, or 3 [9], having well-maintained oral hygiene, with aesthetic concern and willing to undergo minor surgical procedures.

The exclusion criteria included patients with any systemic disease associated with pathological hyperpigmentation or improper delayed wound healing (uncontrolled diabetes, autoimmune diseases, etc.), with nontreated periodontal disease, gingival recession, fenestration and dehiscence, moderate and severe gingivitis cases, chronic smokers, and noncompliant patients.

Stoppage of spicy, acid, colouring and hard food was recommended preoperatively.

### 2.2. Study Design and Participants

**Figure 1:**
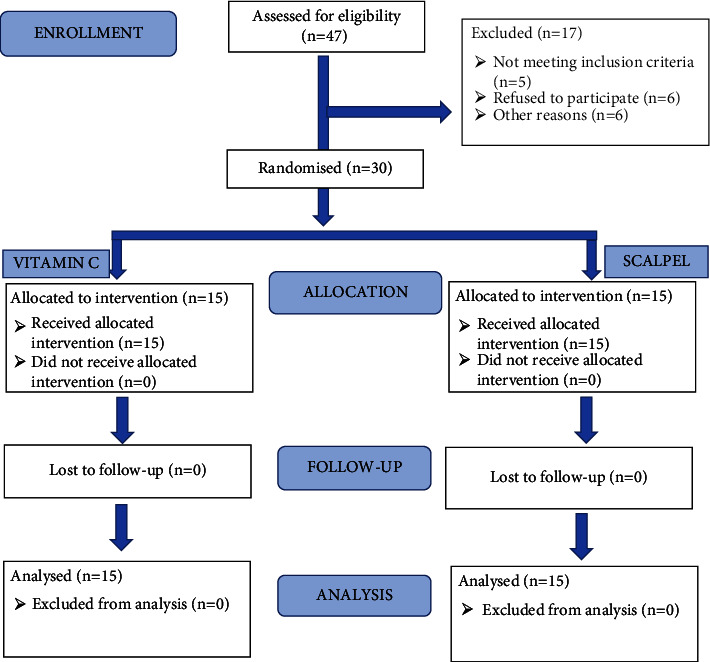
The CONSORT flowchart.

Patients were randomly divided into two groups by lottery method (Figure 1). Group A consisted of 15 samples and gingival depigmentation was done with scalpel technique (control group). Group B consisted of 15 samples, and gingival depigmentation was done with ascorbic acid (test group). Full mouth supragingival and subgingival scaling was done. Patients were educated and motivated to maintain good oral hygiene. An intraoral clinical photograph was taken at baseline before the beginning of the procedure.

Depigmentation was carried out with both techniques and scores were evaluated one week postoperatively after the completion of each procedure. Repigmentation was evaluated at three months.

### 2.3. Depigmentation Procedure

It was carried out on hyperpigmented gingiva in the maxillary or mandibular aesthetic areas, from the canine/1^st^ premolar region to contralateral similar tooth region, with either a scalpel (control group) or vitamin C mesotherapy (test group) procedure. Control and test groups were in opposing sextants so as to avoid bias of results, which could occur by the diffusion of Vitamin C in the neighbouring areas.

**Figure 2:**
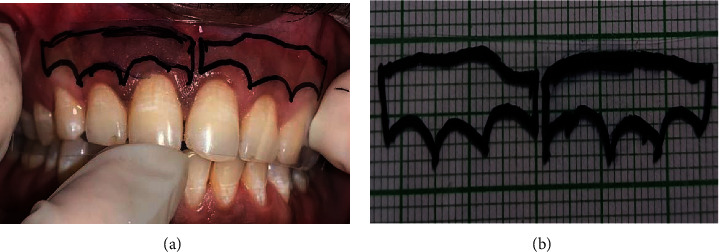
Intraoral marking of gingival hyperpigmentation on cellophane sheet (a); transfer of cellophane sheet on graph paper (b).

The area of pigmentation was measured with a cellophane sheet; which was transferred to the graph paper in order to count the square boxes of one mm calibration (Figure 2).

The visual analogue scale was measured to determine pain scores at 24 hours for both techniques. It was divided into ten numerical parts consisting of one cm each on a horizontal line with two end points (left and right), with “0” representing “no pain” to “10” representing “severe pain.”

A verbal scale for wound itching was measured with a scale with five scores: no itching (0), mild itching (1), moderate itching (2), severe itching (3), and extremely severe (4).

The formula applied for the total area of repigmentation was

(1) $$\begin{array}{c} A=n\times \frac{100}{N}, \end{array}$$

where

- *A* is the percentage area of repigmentation
- *N* is the area in number of squares preoperatively
- *n* is the area in number of squares postoperatively

### 2.4. Surgical Procedure

#### 2.4.1. Scalpel Technique (Group A)

**Figure 3:**
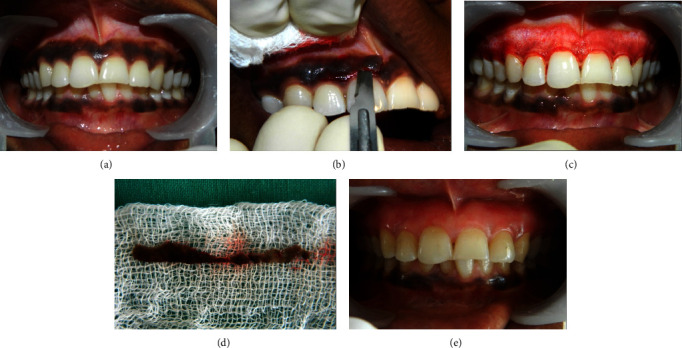
Scalpel technique (control group); preoperative view (a), intraoperative view (b), one week postoperative view (c), excised tissue (d), and three months postoperative view (e).

Local anaesthesia was obtained with infiltration (2% lidocaine with adrenaline 1 : 200.000) in relation to the surgical site. The gingival epithelium was excised with Bard Parker blade number s15 and 11. Partial thickness incision involved the entire hyperpigmented area on the keratinised gingival surface, with the blade placed parallel to the long axis of the teeth, and care was taken not to sever the junctional epithelial attachment to the teeth and also not to expose the underlying bone. Eyelet curette was used to remove the residual hyperpigmented epithelium in the isolated areas. This was followed by careful examination of the exposed connective tissue surface, and the wound was irrigated with saline. Bleeding was controlled using a pressure pack, and once hemostasis was achieved, the site was covered by a periodontal dressing for a period of one week (Figure 3).

#### 2.4.2. Vitamin C Mesotherapy (Group B)

**Figure 4:**
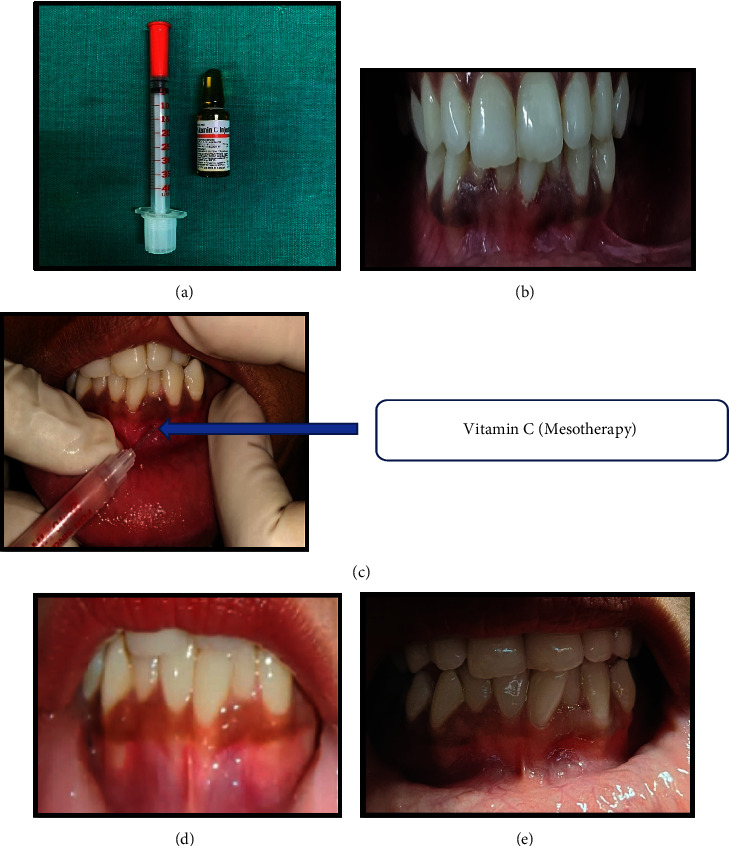
Vitamin C mesotherapy (test group); vitamin C injected with insulin syringe (a), preoperative view (b), intraoperative view (c), one month postoperative view (d), and three months postoperative view (e).

The area was anesthetized by a topical anesthetic agent. Vitamin C ampule of 2 ml about 0.1-0.2 ml (200–300 mg Vitamin C) was injected at approximately epithelial connective tissue interface into the gingiva till the tissue blanch, 2-3 mm apart, using an insulin syringe (needle–30 gauge and 8 mm length). The insulin syringe was introduced into the respective gingival tissues with the bevel facing in the upward direction. This dose of Vitamin C was continued for four weeks, once per week. Follow-up evaluation was carried out at each visit for four weeks, and gingival depigmentation was scored at the one week follow-up after the completion of the procedure. Repigmentation was evaluated at three months. The intensity and duration of itching was assessed at the second visit and the dose was adjusted accordingly. Hence, the dose was used in range of the proposed efficient one, as it varies from individual to individual (Figure 4).

## 3. Results

Thirty patients, 18 females (60%) and 12 males (40%) in the age range of 18–40 years, completed the entire study period. For both the groups, evaluation and comparison of the following parameters were done:

1. VAS scores pain at 24 hours
2. Verbal Itching scores at 24 hours
3. Depigmentation scores after one week postcompletion of each technique
4. Repigmentation at three months

Phases of color changes during vitamin C injection are as follows:

1. Immediately following injection; color of the gingival tissues lightened.
2. Phase 1 (1st week): fading of gingival hyperpigmentation and gingiva became shiny and pulled. Less pigmented areas turned pale pink.
3. Phase 2 (2nd week): more fainting action appeared in relation to the whole gingival tissues and the pinkish color began to intersperse between melanin pigmented areas.
4. Phase 3 (3rd week): color of melanin pigment reduced, pink color increased.
5. Phase 4 (4th week): near normal gingival color seen.

### 3.1. Statistical Analysis

The sample size was calculated using G Power software from the data obtained from a previous study conducted by Narendra et al. [1]. Gingival hyperpigmentation scores were measured preoperatively. Depigmentation, pain, itching, and repigmentation scores were assessed postoperatively. Descriptive statistics are expressed as mean ± standard deviation (SD). For comparing the area of pigmentation, an independent *t*-test was applied and Mann–Whitney *U* test was used for differentiating the intensity of pigmentation, repigmentation, and VAS score between the groups (Table 1).

**Figure 5:**
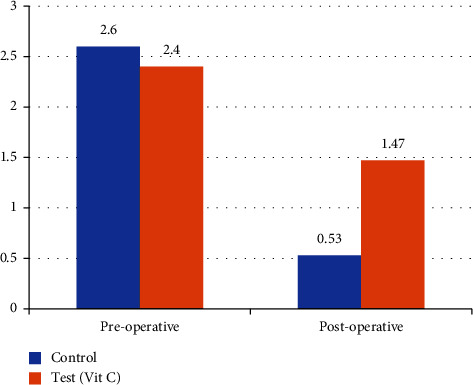
Comparison of intensity of pigmentation (mean gingival pigmentation index [9]) between the test and control group.

There was no statistically significant difference in preoperative intensity of pigmentation between the test and control group (*p*=0.076). Similarly, postoperatively also, there was no statistically significant difference in intensity of pigmentation (*p*=0.754) between test and control group (Figure 5).

**Figure 6:**
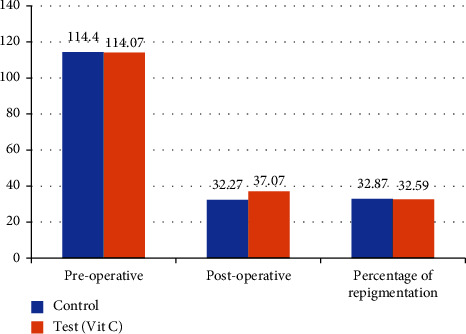
Comparison of area of pigmentation (in mm^2^) between the test and control group.

Likewise, there was no statistically significant difference in preoperative area of pigmentation between the test and control group (*p*=0.936). Postoperatively, there was no statistically significant difference in area of pigmentation between the test and control group (*p*=0.932). There was no statistically significant difference in percentage of repigmentation between the test and control groups (*p*=0.903) at the end of three months (Figure 6).

**Figure 7:**
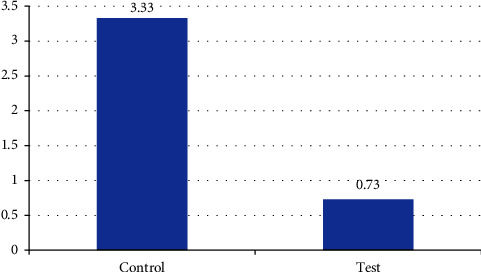
Mean VAS score for pain.

After 24 hrs, the test group showed significantly lesser VAS score for pain as compared to the control group (*p*=0.001, Figure 7).

None of the participants reported with itching after vitamin C mesotherapy. Clinically, there was less reduction in intensity of melanin pigmentation with the test group compared to the control group.

## 4. Discussion

Gingival depigmentation has become an aesthetic concern in the present era. Myriad methods like surgical and nonsurgical have been successfully employed regarding treatment of the gingival pigmentation [1, 10–12]. Despite the various available techniques, depigmentation with a scalpel remains the gold standard. However, there are several disadvantages associated such as fear of surgical blade, pain, bleeding, healing by secondary intention, and an open wound site [3, 9, 13, 14].

Vitamin C is a potent antioxidant as well as a depigmenting agent that has been successfully used intradermally [15], intramucosally, as well as topically [3, 16, 17]. The mechanism of action of Vitamin C is that it interacts with copper ions at the tyrosinase active site and inhibits its action, leading to a decrease in the formation of melanin. Ascorbic acid also helps in the postoperative healing of tissues by aiding in collagen synthesis, differentiation, and maturation of fibroblasts [18, 19].

Vitamin C, although minimally invasive, has certain limitations, and precautions have to be taken to avoid complications. It is a weak acid and if given in excess may cause necrosis of the underlying gingival tissues. In addition, there are certain critical areas which should be taken care off while using vitamin C injections, such as the interproximal gingiva, the mucogingival junction, and areas of thin gingival phenotype [20].

The present study compared areas and intensities of preoperative and postoperative pigmentation within and between the groups. VAS scores were evaluated for comparison of postoperative pain between the groups, and repigmentation was assessed between the groups after three months.

Results of the present study showed that statistically comparable outcomes can be achieved with both techniques. However, clinically, certain observations attract our attention:

1. Potency of melanin pigmentation reduction is far better with scalpel technique
2. Very darkly pigmented gingiva should not be taken up for Vitamin C intramucosal injections
3. Pain scores were very less with ascorbic acid treatment
4. Depth of penetration cannot be controlled with Vitamin C
5. Multiple appointments and punctures with Vitamin C therapy can be a disadvantage

Gingival repigmentation after depigmentation is one of the reported factors. It refers to the clinical appearance of melanin pigment following a period of clinical depigmentation [21]. Repigmentation depends on a myriad of factors. It is predicted that gingival repigmentation occurs as a result of the migration of neighbouring melanocytes in the surgical area [22]. Recurrence associated with surgical depigmentation is more common in thin periodontal phenotype [20, 23]. Repigmentation is also dependent upon confounding factors like smoking, sun exposure, the genetic code of the skin, technique used, and the duration after which pigmentation is measured.

Our trial showed outcomes in accordance with the study done by Yussif et al. [3], which revealed equivalent and significant improvement in gingival color at four weeks with scalpel and Vitamin C mesotherapy.

Sheel et al. [24] have reported satisfactory aesthetic results after gingival depigmentation with a scalpel along with local applications of ascorbic acid, with resultant lesser pain scores and no repigmentation areas at nine month follow-up.

Dadlani et al. [25] compared depigmentation using a scalpel and a diode laser and have reported that both the scalpel and the laser were efficient when used efficaciously for gingival hyperpigmentation.

Our trial showed approximately 33% of repigmentation scores with both techniques after three months. Probably, a combination of both techniques and a larger sample size with a longer follow-up period may give a better insight to advantages and disadvantages of individual and combined techniques.

Future advancements for the gingival hyperpigmentation treatment techniques can be the inclusion of plasma therapy [26]. Plasma therapy acts by release of free radicals and reactive oxygen species and production of ozone [27].

Another novel technique proposed for gingival depigmentation is the utilization of radiofrequency waves. The latent heat produced slows the emigration of melanocytes which reside in the basal and suprabasal layers of gingival epithelium [28].

From the perspective of the authors, the technique for gingival hyperpigmentation to be chosen depends on the periodontal phenotype, intensity of gingival hyperpigmentation, patient selection, and compliance. Furthermore, it can be vocalized that long-term studies are needed in regards to Vitamin C mesotherapy techniques.

### 4.1. Limitations

1. In the present study, the term “periodontal phenotype” is just used as a relative term, not comparing the gingival thickness by any quantitative method. More accurate measurements of the periodontal phenotype can be done by various methods like: CBCT [29], periodontal probe visibility [30], measurement with the help of endodontic reamer [31] and numerically i.e. gingival thickness <0.8 mm being thin phenotype and more than the same numerical value termed as thick phenotype [32].
2. Maxillary and mandibular gingival sextants were randomly treated by either scalpel or mesotherapy techniques, which can be a limiting factor of the study, as maxillary gingiva has a relatively thick phenotype as compared to that of the mandibular region.

## 5. Conclusion

Vitamin C mesotherapy and scalpel technique showed no statistically significant differences in reduction of areas and intensity of depigmentation (*p* > 0.05) (Dummett and Bolden index [9]) and repigmentation (*p* > 0.05), therefore, Vitamin C mesotherapy proved to be as effective as the conventional surgery. Vitamin C out-matched scalpel technique with reduced VAS scores for pain and better patient acceptance but estimation of depth of depigmentation locus and completeness of the procedure in terms of intensity reduction were characteristic features of scalpel surgery. However, a larger sample size, the combination of both procedures, and a longer follow-up period may throw a better insight towards the actual outcome of our study.

## Figures and Tables

**Table 1:** Comparison of intensity, area of pigmentation, repigmentation, and pain score between the test and control group.

Parameters	Groups	*N*	Mean ± SD	Difference	*p* values
Preoperative intensity	Control	15	2.60 ± 0.51	0.20	0.076 (NS)
	Test	15	2.40 ± 0.63		

Postoperative intensity	Control	15	0.53 ± 0.52	−0.94	0.754 (NS)
	Test	15	1.47 ± 0.64		

Preoperative area of pigmentation	Control	15	114.40 ± 8.72	0.33	0.936 (NS)
	Test	15	114.07 ± 13.24		

Postoperative area of pigmentation	Control	15	32.27 ± 6.67	0.20	0.932 (NS)
	Test	15	37.07 ± 5.99		

Percentage of repigmentation	Control	15	32.87 ± 7.04	0.28	0.903 (NS)
	Test	15	32.59 ± 5.14		

VAS score	Control	15	3.33 ± 0.82	2.60	0.001^*∗*^
	Test	15	0.73 ± 0.71		

NS: nonsignificant difference at *p* ≤ 0.05; ^*∗*^indicates significant difference at *p* ≤ 0.05.

## Data Availability

The data used to support the findings of this study are available from the corresponding author upon request.
